# Supplementary material for: A Putative Lignin Copper Oxidase from Trichoderma reesei
Source: J Fungi (Basel). 2021 Aug 7;7(8):643. doi: 10.3390/jof7080643 (PMC8400822; doi:10.3390/jof7080643)
Supplement: Supplementary file 1 [file jof-07-00643-s001.zip › jof-1312490-supplementary.pdf]

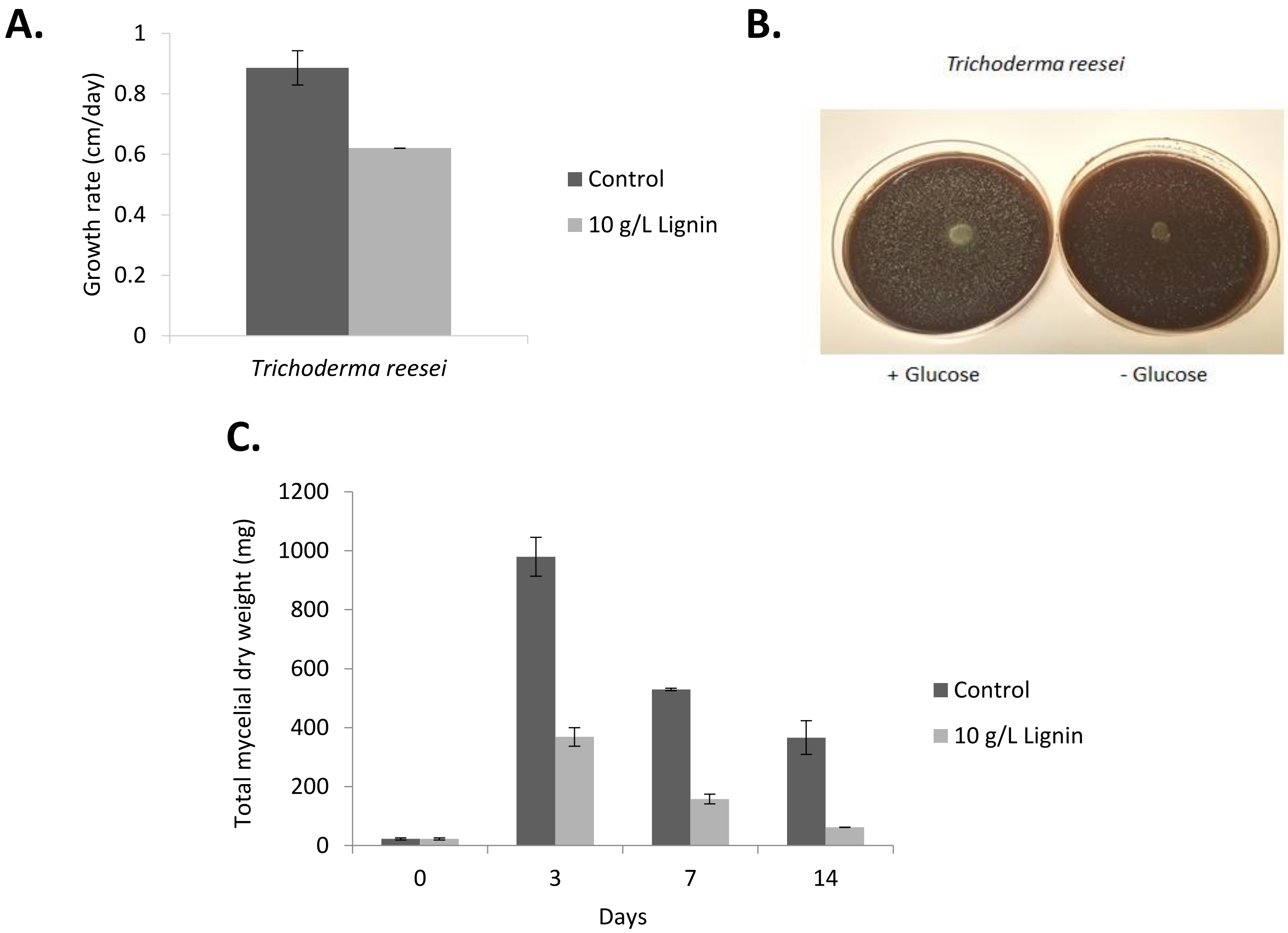

Figure S1. *T. reesei* growth on technical soda lignin. A. Growth rate of *T. reesei* on agar plates containing technical lignin in the presence and absence of 1 g/L glucose and **B.** mycelial appearance. **C**. Total mycelial dry weight of *T. reesei* during growth in liquid cultures on lignin in the presence and absence of 2.5 g/L maltose.

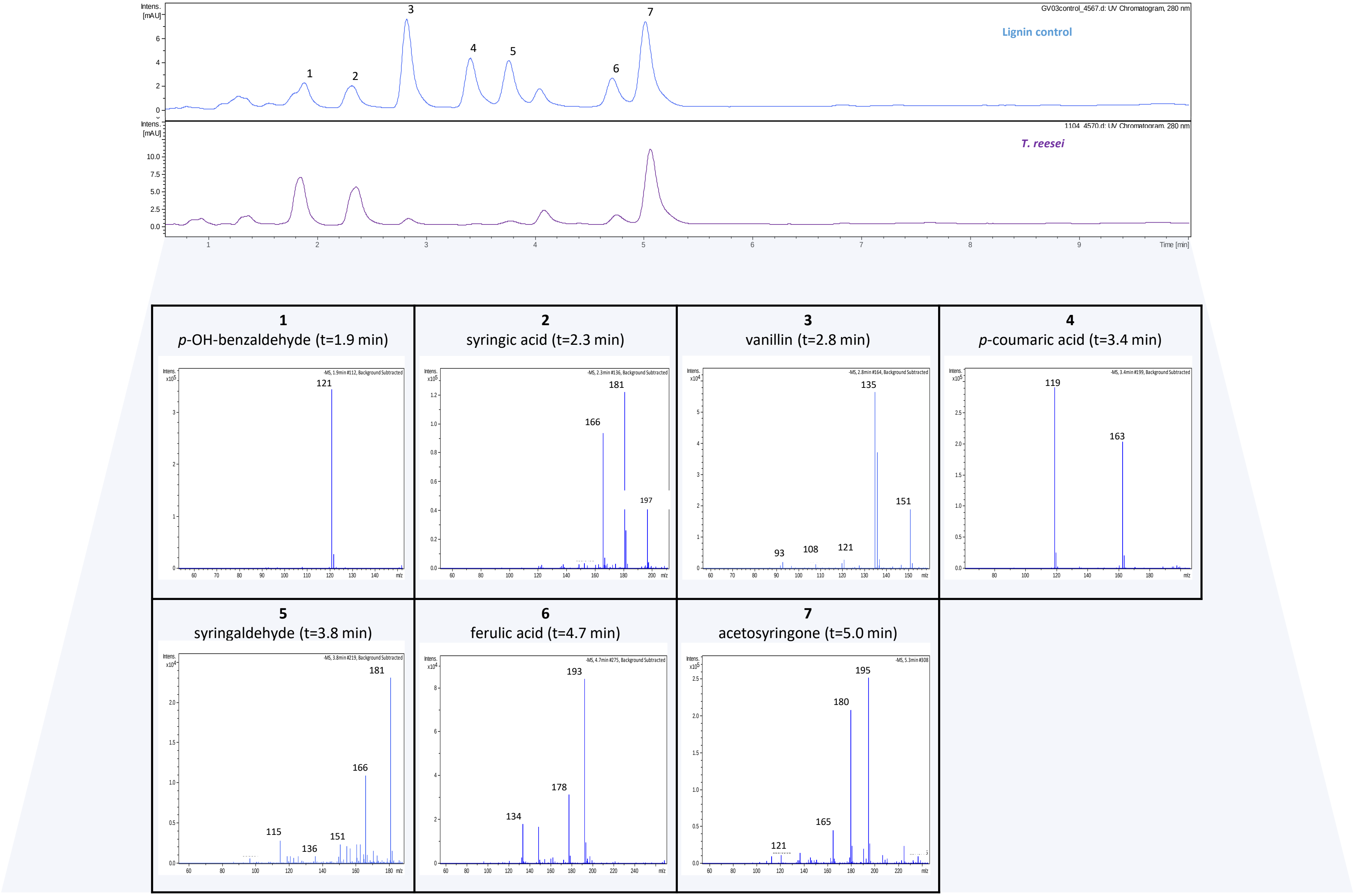

Figure S2. LC-MS analysis on water-soluble lignin fraction. The analysis is done on phenolic monomers extracted from the culture supernatant by ethyl acetate. Normalized chromatograms obtained with a C18 column (Highpurity, Thermo Electron Corporation, 2.7 µm, 50 mm x 2 mm I.D.mm), a 5–100 vol.% aqueous acetonitrile, 1‰ HCOOH gradient (30min) and 0.4 ml/min flow rate, and with a 280 nm UV detection. ESI-MS spectra were obtained in the negative mode from scans acquired in a mass range of m/z 120–2000.

Table S1. **Total phenolic and thioacidolysis yields of water-insoluble residual lignin after exposure to *T. reesei*.**

	**PheOH (mmol g^{-1})[a]**	**Thioacidolysis[b]**	
		**Total yield (µmol g^{-1})**	**S/G ratio**
**Control**	2.90	132 ± 9	0.93 ± 0.05
T. reesei	2.80	89 ± 3	0.92 ± 0.00

[a] Data correspond to single determination

[b] Data are mean values (± standard error) between duplicate analyses.

Table S2. Identified CAZymes in the secretomes during growth of *T. reesei* on technical lignin.

Protein ID	Cazy	Day of growth	Protein ID	Cazy	Day of growth
22915	AA3_2	7	69276	GH30_7	7 and 14
80659	AA3_3	14	82235	GH31	3, 7 and 14
**124282**	**AA5_1**	**3, 7 and 14**	80240	GH35	3, 7 and 14
78357	AA6	7 and 14	124016	GH36	3, 7 and 14
73643*	AA9-CBM1	7	123226	GH37	3, 7 and 14
77299	GH2	3, 7 and 14	3196	GH38	14
69245	GH2	3, 7 and 14	68064	GH43	7
62166*	GH2	7	45717	GH47	3, 7 and 14
58450	GH3	3, 7 and 14	55319	GH54-CBM42	3, 7 and 14
121735	GH3	3, 7 and 14	123283	GH54-CBM42	3, 7 and 14
121127	GH3	3, 7 and 14	121746	GH55	3, 7 and 14
47268	GH3	7	54242	GH55	3, 7 and 14
76672	GH3	7 and 14	70845	GH55	3, 7 and 14
104797	GH3	7 and 14	73248	GH55	3, 7 and 14
82616*	GH5_5	7	76210	GH62	3, 7 and 14
120312	CBM1-GH5_5	7	124175	GH64	3, 7 and 14
56996	GH5_7	7	123456	GH65-CBM32	3, 7 and 14
72567	CBM1-GH6	7 and 14	72526	GH67	3, 7 and 14
123989	GH7-CBM1	3, 7 and 14	71532	GH71	3, 7 and 14
122081	GH7-CBM1	7 and 14	108672	GH71-CBM24-CBM24	3, 7 and 14
120229*	GH10	7	120873	GH71-CBM24-CBM24	3, 7 and 14
74223	GH11	7 and 14	123538*	GH72	7
123818	GH11	3, 7 and 14	22914	GH72-CBM43	3, 7 and 14
123232*	GH12	7	82633	GH72-CBM43	3, 7 and 14
105956	GH13_1	3, 7 and 14	49081	GH74-CBM1	3, 7 and 14
1885	GH15-CBM20	3, 7 and 14	70341*	GH75	7
65406	GH16	3, 7 and 14	74807*	GH76	7 and 14
123726	GH16	3, 7 and 14	27395	GH76	7
49274	GH16	3, 7 and 14	67844*	GH76	7
39755	GH16	7	106575	GH79	3, 7 and 14
76266*	CBM18-GH16	7	71394	GH79	7
66792	GH17	3, 7 and 14	57098	GH92	3, 7 and 14
39942	GH17	3, 7 and 14	55733	GH92	3, 7 and 14
80833	GH18	3, 7 and 14	60635	GH92	7 and 14
119859	GH18	7 and 14	79921*	GH92	7
68347	GH18-CBM1	7 and 14	58802	GH95	3, 7 and 14
21725	GH20	3, 7 and 14	111138*	GH95	7
23346	GH20	7	5807*	GH95	7
103458	GH25	7	79606	GH115	7 and 14
72632	GH27	3, 7 and 14	70373	GH125	7 and 14
59391	GH27	3, 7 and 14	107850	CE1	7
27259*	GH27	7	44214	CE5	3, 7 and 14
122780	GH28	3, 7 and 14	73632*	CE5-CBM1	3 and 7
103049	GH28	7 and 14	58282	CE9	14
3094	GH30_3	3, 7 and 14	121418	CE16	3, 7 and 14
69736	GH30_3	7 and 14	103033	PL7_4	7
110894	GH30_5	3, 7 and 14	69189*	PL20	7
111849	GH30_7	3, 7 and 14			

*proteins that were exclusively detected in the absence of maltose.

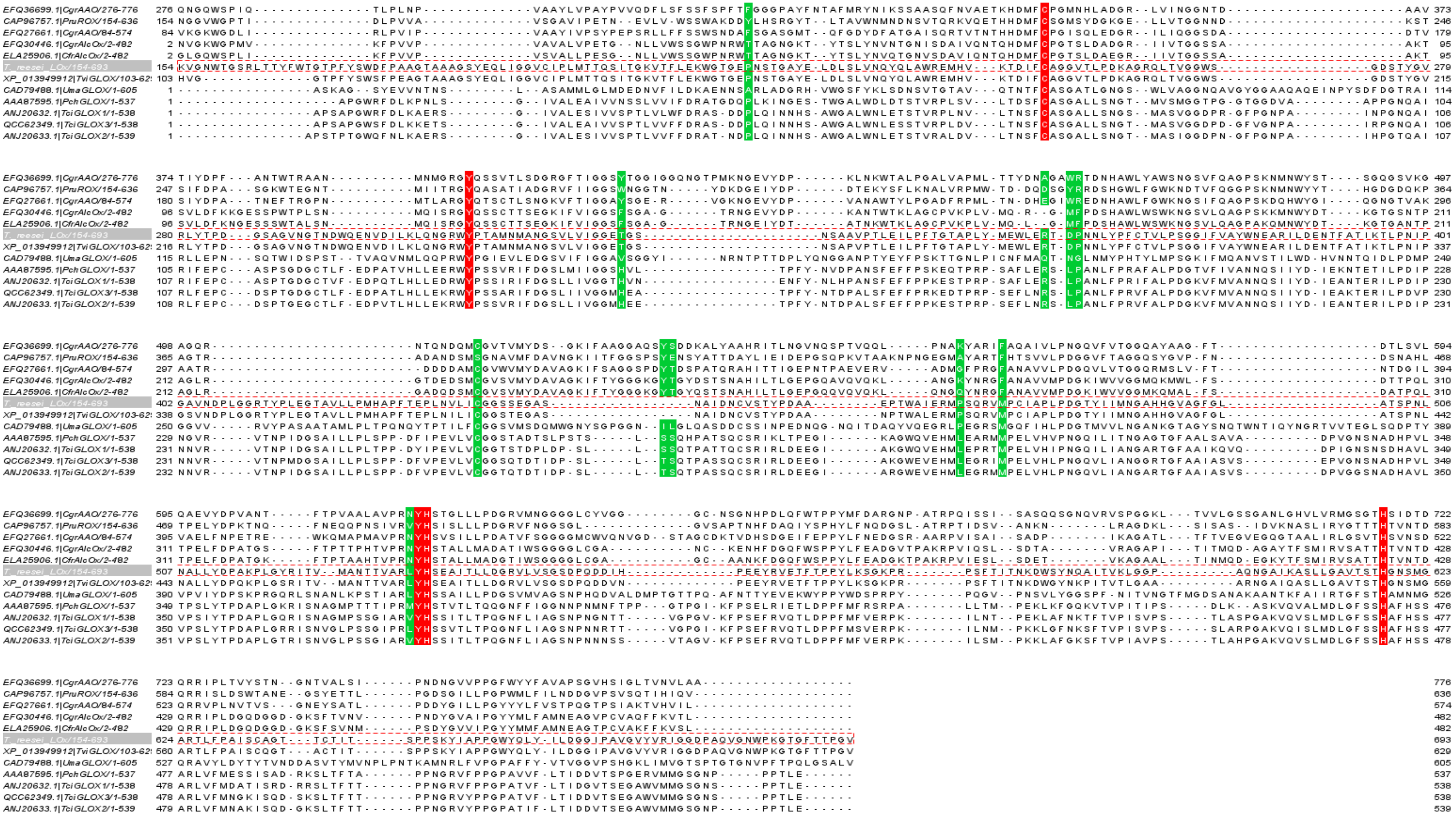

Figure S3. Sequence alignment of 11 functionally characterized AA5 enzymes with *Tr*LOx. EFQ36699.1: aryl alcohol oxidase from *Colletotrichum graminicola*; CAP96757: Raffinose oxidase from *Penicillium rubens*; EFQ27661: aryl alcohol oxidase from *C. graminicola*; EFQ30446: Alcohol oxidase from *C. graminicola*; ELA25906: Alcohol oxidase from *C. fructicola*; XP_013949912: Glyoxal oxidase from *T. virens*; CAD79488: Glyoxal oxidase from *Ustilago maydis*; AAA87595: Glyoxal oxidase from *Phanerochaete chrysosporium*; ANJ20632: Glyoxal oxidase 1 from *Trametes cinnabarina*; QCC62349: Glyoxal oxidase 3 from *T. cinnabarina*; ANJ20633: Glyoxal oxidase 1 from *T. cinnabarina.* Residues involved in copper binding are highlighted in red and other residues involved in enzymatic activity and substrate preference are highlighted in green.

A.

B.

C.

D.

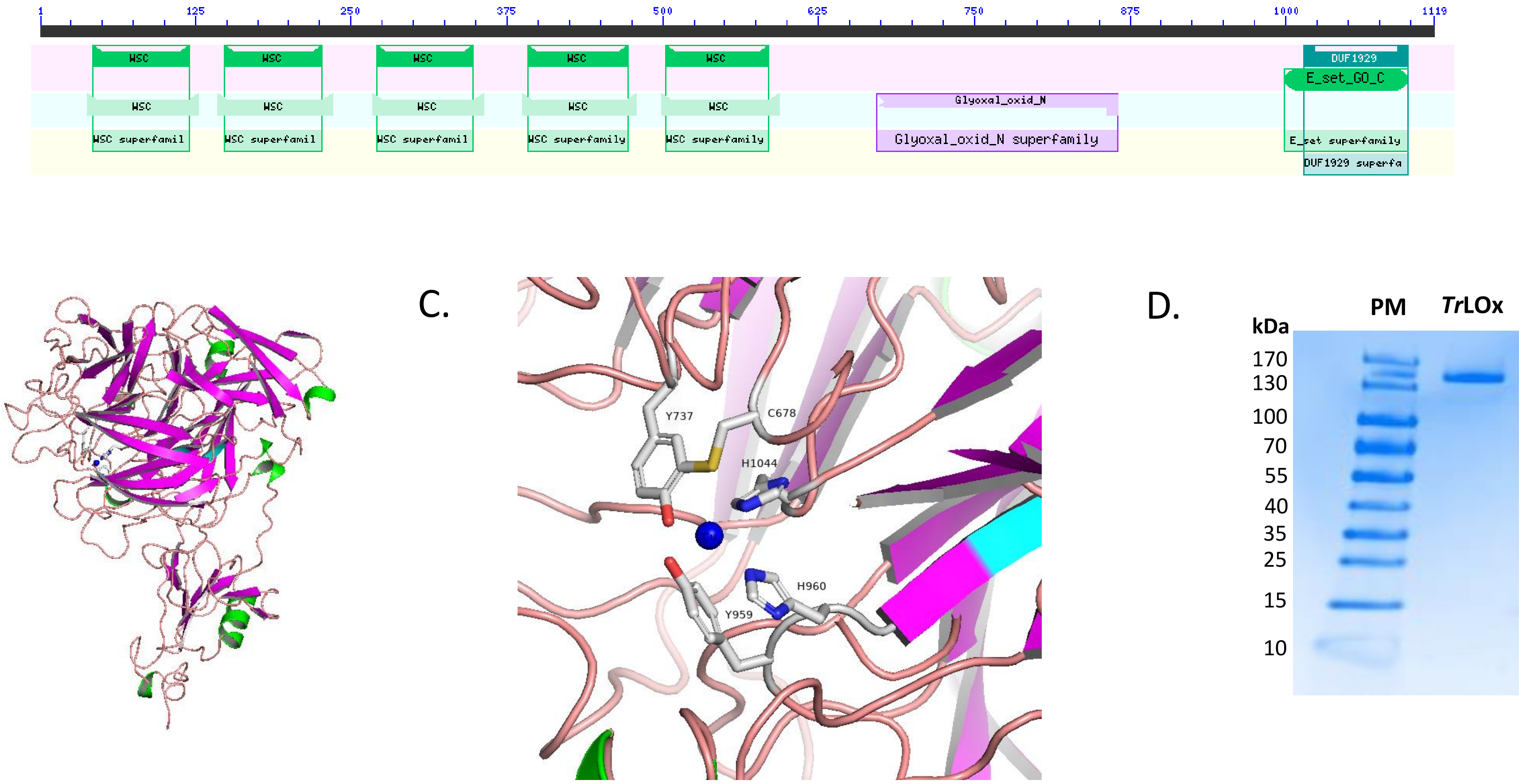

Figure S4. Structural characteristics of *Tr*LOx. A. Predicted domain and functional sites in *Tr*LOx encoding sequence. WSC domain: wall stress-responsive component; E_set_GO_C: C-terminal Early set domain associated with the catalytic domain of galactose oxidase at the C-terminal end; DUF1929: Domain of unknown function. **B.** Predicted molecular structure of *Tr*LOx. The model was generated using Phyre2 and with copper oxidase from *Colletotrichum graminicola* as template (PDB 6RYX). **C.** The active site of *Tr*LOx containing the aromatic residues implicated in copper coordination. **D.** SDS-PAGE gel of purified *Tr*LOx protein.

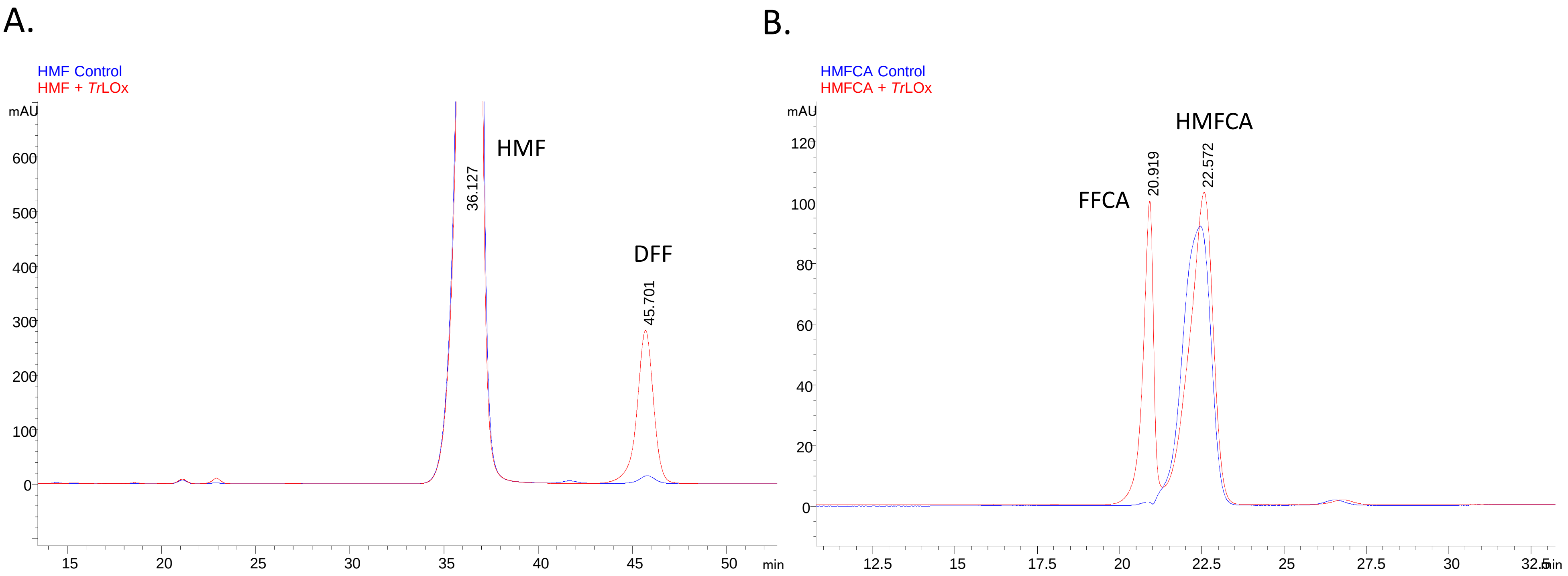

Figure S5. HPLC analysis of *Tr*LOx reactions on A. HMF and B. HMFCA. The reactions were performed at 30 °C under agitation at 800 rpm over 24 h period in 50 mM tartrate buffer pH 6 and in the presence of 10 µg of enzyme and 5 mM of substrate. Reaction mixtures were separated on Aminex HPX87H column (300 × 7.8 mm) (BioRad) at 45 °C, with 2.5 mM sulfuric acid as the isocratic mobile phase with a flow rate of 0.6 mL/min. Eluted compounds were detected using a diode array detector at 280 nm. The reactions were stopped by incubating the mixture at 90 °C for 10 min and centrifuging at 15,000×g for 15 min. In blue are HMF and HMFCA standards and in red is the reaction with *Tr*LOx.

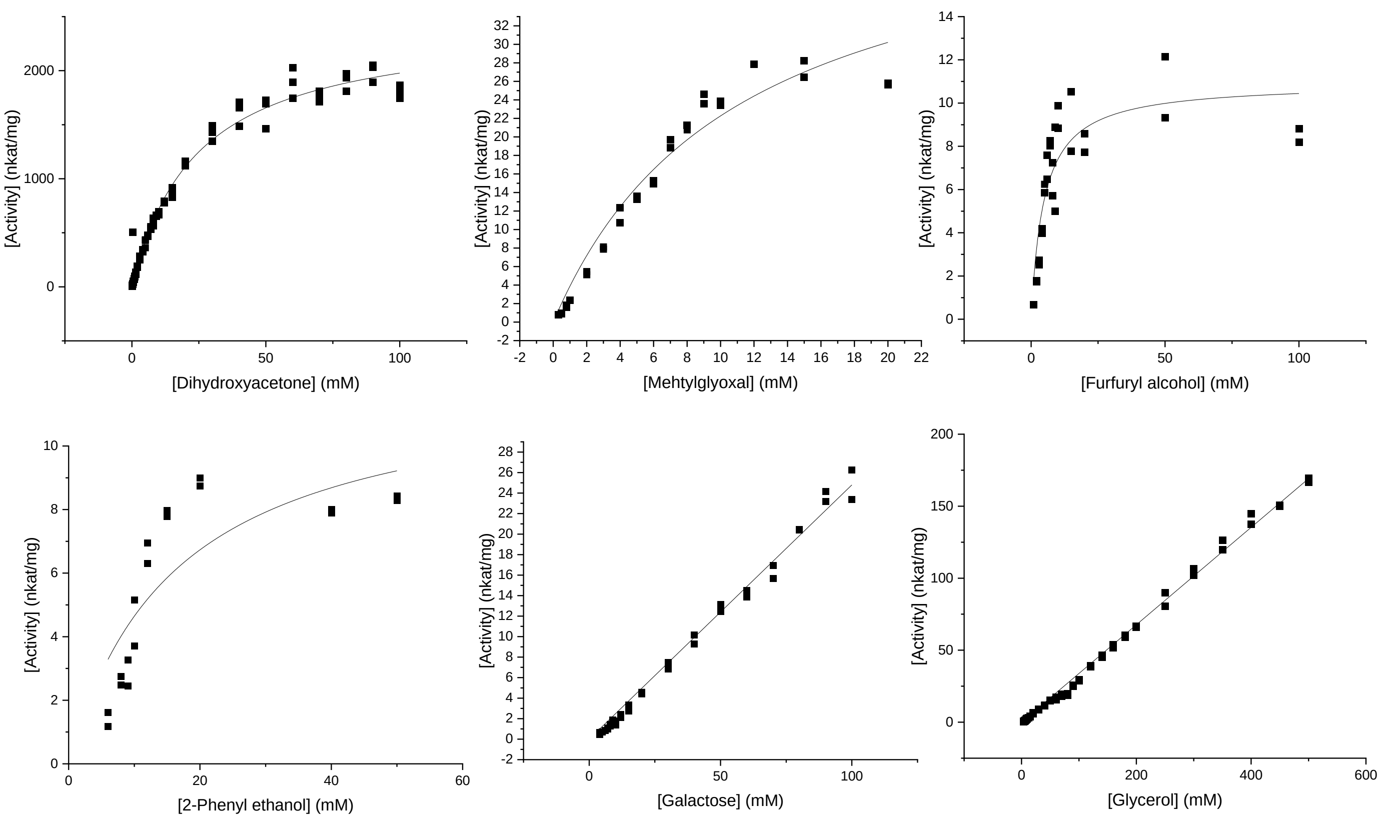

Figure S6. Michaelis–Menten plots for the activity of *Tr*LOx on the different tested substrates.

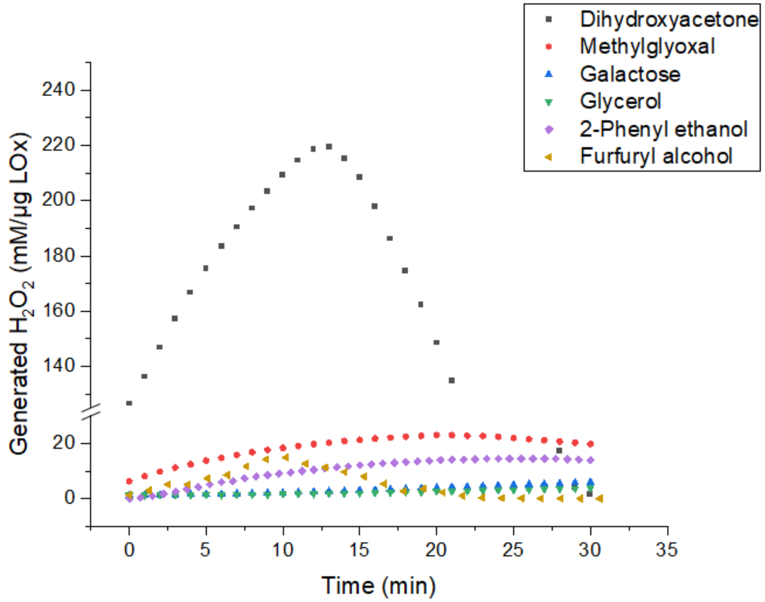

Figure S7. H_2O_2 production overtime in the reaction of *Tr*LOx on different substrates. The fluorescence was followed at an excitation wavelength of 560 nm and an emission wavelength of 595 nm. The slope from the standard curve relating H_2O_2 concentration and fluorescence was used to calculate the amount of generated H_2O_2 over time (0-20 µM H_2O_2; 374.34 counts/µmol).

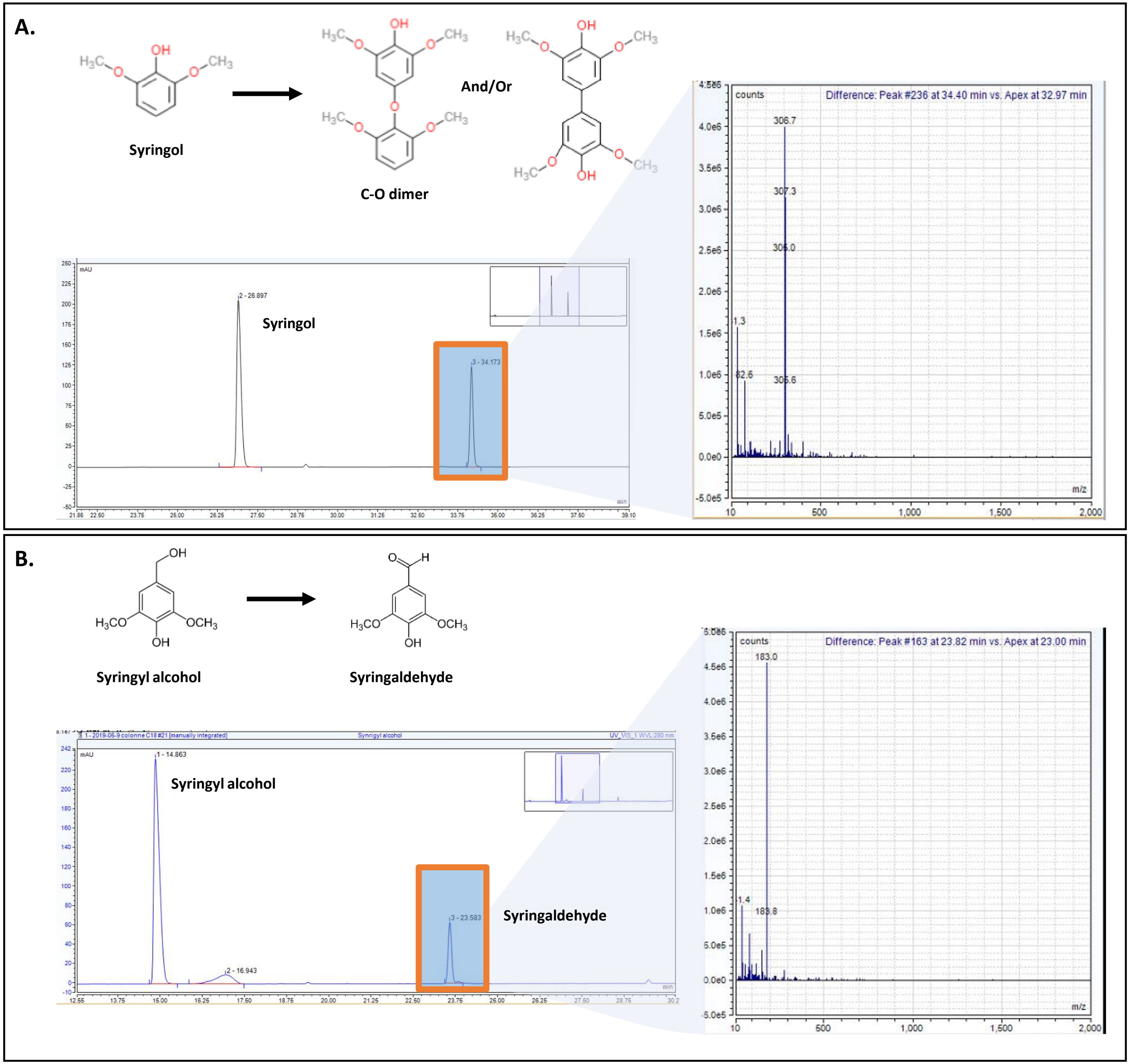

Figure S8. LC-MS chromatograms of the reaction of *Tr*LOx on A. Syringol and B. Syringyl alcohol. The molecular weight of the detected product with syringol (306.7) suggest the formation of C-C and/or C-O dimers.
